# Health promoting behaviors in low-income overweight and obese women in Korea: an exploratory qualitative study

**DOI:** 10.4069/kjwhn.2021.11.30

**Published:** 2021-12-13

**Authors:** Ju-Hee Nho, Eun Jin Kim

**Affiliations:** 1 College of Nursing, Jeonbuk Research Institute of Nursing Science, Jeonbuk National University, Jeonju, Korea; 2 College of Nursing, Jeonbuk National University, Jeonju, Korea

**Keywords:** Income, Life style, Obesity, Qualitative research, Republic of Korea, Women

## Abstract

**Purpose:**

This study aimed to explore and understand the health promoting behaviors of low-income overweight and obese women in Korea.

**Methods:**

Data were collected from 10 low-income overweight and obese women working at a community self-sufficiency center through semi-structured in-depth interviews. Individual interviews were conducted and transcribed. Deductive content analysis was done, using the MAXQDA program.

**Results:**

The health promoting behaviors practiced by low-income overweight and obese women were affected by intrapersonal, interpersonal, and organizational/community factors. Six categories were identified and two category clusters were derived that could best describe their health promoting experiences. As main category clusters, despite “feeling that the body and mind are not healthy” participants noted “difficulty maintaining a healthy lifestyle.” Overall, the participants had poor nutritional status, lacked physical activity, experienced much stress in intrapersonal level, and faced intrapersonal-level barriers to health promoting behaviors. Moreover, participants had a lack of personal will, and lack of specific information to practice health promoting behaviors, a lack of time, and too many overall burdens to earn a living for their family while trying to maintain health promotion behaviors.

**Conclusion:**

Lifestyle interventions for nutrition management, encouragement of physical activity, and stress management are needed for overweight and obese low-income women. In addition, social support and policies are needed to improve their living environment.

## Introduction

Obesity can cause diseases such as type 2 diabetes, dyslipidemia, high blood pressure, and fatty liver [1]. According to the 2019 Korean National Health and Nutrition Examination Survey (KNHNES) report the obesity rate of Korean adults over age of 19 years increased from 31.9% in 2011 to 34.4% in 2019, while the obesity rate among women over the age of 30 years increased from 25.7% in 2011 to 27.4% in 2019 [2]. Obesity is also associated with socioeconomic factors [3]. In the United States, more than 30% of adults are obese. Regarding income, the prevalence of obesity was significantly higher among the low-median income household and low-education black (34.2%) and Hispanic populations (31.7%) compared to whites (21.9%) [3]. The prevalence of obesity over the age of 19 years in Korea is 34.4%. When classified by income level, 35.6% of the low-income group compared to 33.7% of the high-income group [2]. This indicates a higher prevalence of obesity in the low-income group than in the high-income group.

Women tend to accumulate fat more easily because of changes in female hormones such as pregnancy, childbirth, and menopause, and because their basal metabolic rate and energy consumption are lower than that of men [4]. The rate of performing aerobic physical activity steadily decreases among women in their 40s (42.9%), 50s (41.9%), 60s (34.5%), and 70s and older (28.6%) [2]. In addition, according to the KNHNES, while there was no correlation between obesity rate and income level for adult males, in adult women, lower education and income levels were associated with higher obesity rate [2]. The obesity rate among Korean women was 6.3 times higher for women with a low education than for those with a higher education, which was the highest among Organization for Economic Co-operation and Development (OECD) countries [2]. In addition, the obesity rate of Korean women in rural areas was 9.3% higher than that in urban areas, and 7% higher at the lower income level than at the upper level [2]. This phenomenon is characteristic of women, unlike men, indicating that the changes in the relationship between income level and obesity are similar to other developed countries. In particular, it suggests the need for health management for low-income obese women.

Low-income women had less access to fresh and healthy food and consumed more sodas, sugary drinks, and fast food [5]. In addition, reports also show that the high obesity rate among low-income women is the result of reduced access to sports facilities and low physical activity [6].

In the low-income class, women are identified as the most representative of health inequalities and have different and comprehensive problems, such as malnutrition and obesity [7]. Low-income women are more likely to eat simple nutrients owing to socioeconomic conditions and no food variety. Therefore, their health practices are lower than those of other classes with higher health risks such as smoking and drinking [8]. As a result, low-income women have many health risk factors and are unable to properly practice health promotion behaviors owing to various factors [7].

According to a study on the difference in health behavior among social classes, high-income women have high rates of health promotion behavior, such as non-smoking, physical activity, and medical checkups, while low-income obese women have poor regular exercise habits and low physical activity [9]. In addition, women tend to prioritize family-related tasks such as housework and childrearing over their own work which can also affect their behavior [10]. As such, some studies confirm the differences and causes of health promotion behavior according to income. It is difficult to find studies on the experiences of health promoting behaviors in low-income overweight and obese women.

Qualitative content analysis is a research method that draws valid inferences by objectively and systematically analyzing research data to understand the phenomenon to be explored. In qualitative content analysis, there are inductive and deductive methods. Among them, deductive content analysis is a useful method for retesting concepts in light of existing theories or hypotheses [11]. In this study, based on the intrapersonal, interpersonal, and organization/community factors suggested by McLeroy et al. [12] in the ecological model, a deductive content analysis was attempted on the health promoting behaviors of low-income, overweight and obese women. The experience of health promoting behaviors of all participants in this study is affected by intrapersonal, interpersonal, and organization/community factors, the deductive content analysis method can be thought to be appropriate.

The purpose of this study is to help understand low-income overweight and obese women and provide basic data for developing related nursing interventions. Therefore, this study aimed to explore how socioeconomically disadvantaged low-income overweight and obese women experience and practice health promoting behaviors. In particular, to understand what is difficult and what is helpful to maintain health promoting behaviors. The research question for this study is "How about health promotion behaviors experienced by low-income, overweight and obese women?’

## Methods

Ethics statement: This study was approved by the Institutional Review Board of Jeonbuk National University (2018-12-007-001). Informed consent was obtained from the participants.

### Research team and reflexivity

#### Researcher characteristics

The research team is comprised of two individuals who work in academia and identify as female. Both have been registered nurses for 13 years (JHN) and 8 years (EJK) in women's health care settings. Interviews were done by an author holding a PhD who has experience or research using qualitative methodology with women (JHN), and the other author holds a master degree (EJK) and participated in workshops and seminars in qualitative research. One researcher (JHN) was employed as professor in women health nursing, and the other researcher (EJK) was research assistant. There was a no relationship established prior to study commencement between researchers and participants.

#### Relationship with participants

Participants did not know about the researcher, but they were informed of the purpose of the interview. A reflection diary was kept with research team members to account for the researcher’s influence on the data. All decisions regarding data collection and analysis were discussed and agreed upon by the team members.

### Study design

This study is a descriptive qualitative study on the experiences of health promoting behaviors of low-income overweight and obese women. Descriptive research is the method of choice when a direct description of a phenomenon is required [13]. This study collected data through in-depth interviews and identified concepts and topics through the collected data. The description of the study was reported according to the Consolidated Criteria for Reporting Qualitative Research [14].

### Participant selection

*Sampling*: Participants were selected via purposeful sampling

*Method of approach*: Study advertisement flyers were posted at a community self-sufficiency center in Jeonju, Korea. interview.

*Sample size/non-participants*: Women aged 20 years or older, with a body mass index (BMI) of 23 kg/m^2^ or more, and earning less than the lower first quartile of household income (average of 1,424,000 [two persons] Korean won for 2019; approximately 1,200 US dollars) were invited to participate in in-depth interviews. Of the 15 women who agreed to the study, 10 remained after excluding four persons with a BMI of less than 23 kg/m^2^ and one who refused to meet for the interview.

### Setting

*Setting of data collection:* Interviews were conducted from August 28 to September 19, 2019, at a community self-sufficiency center in Jeonju, which helps the underprivileged who have difficulty finding work opportunities to work on their own and supports the conditions for stable economic activity.

*Presence of non-participants*: There was nobody besides interviewers and participants during the interview.

*Description of sample*: The mean (±standard deviation) age of the participants was 42.6 (±13.9) years with half in their 50s (n=5), and the mean BMI was 30.5 kg/m^2^ (range, 23.8–40.6 kg/m^2^). All participants had a monthly household income of less than 1 million Korean won (roughly 900 US dollars), which was worse than the income level of the inclusion criteria (Table 1).

### Data collection

Individual face-to-face in-depth interviews were conducted in a quiet meeting room in the community self-sufficiency center at a time convenient for the participants. All interviews started with the open-ended question, the main question was, “Please tell me about the health promoting behaviors experienced by the participants.” Encourage participants to talk about the topic as freely as possible and for other questions, a list of auxiliary questions was prepared and the interview was conducted. The list of auxiliary questions was prepared based on the intrapersonal, interpersonal, and organization/community factors aspects in the ecological model [12].

Each interview lasted an average of 60 minutes (ranging from 40 to 90 minutes) and each participant was interviewed once or twice.

All interviews were recorded with separate consent of participants and transcribed verbatim immediately. In addition, nonverbal responses such as facial expressions and movements of the participants were noted in field notes and used for analysis. Interviews and analysis were performed simultaneously and saturation of the data was determined when new data were no longer available.

### Data analysis

All authors participated in data coding. The MAXQDA program (VERBI GmbH, Berlin, Germa­ny) was used for content analysis.

**Figure 1.**
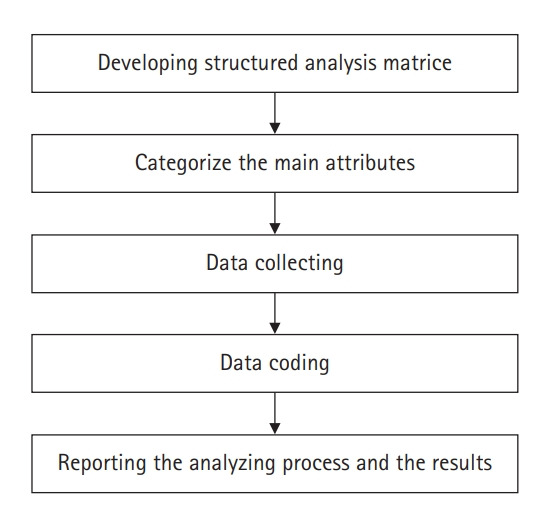
Process of content analysis in this study.

*Derivation of themes*: This study was analyzed via content analysis [11] (Figure 1). In the preparatory stage, it was decided to analyze the health promotion experiences of low-income, overweight and obese women as the subject of the study, based on the intrapersonal, interpersonal, and organizational/community factor aspects in the ecological model [12], the following five-step process was performed to confirm health promoting behaviors attributes through a deductive method. The first stage is to analyze the existing literature and develop a categorization matrix. In the first stage, a categorization matrix was developed based on intrapersonal, interpersonal, and organizational/community factors for health promotion progress based on the ecological model [12]. The second step is to categorize the main attributes. Through the developed categorization matrix, the main categories and sub-areas were set as initial coding categories and categorized, an operational definition was given to each category. As suggested by McLeroy et al. [12], the intrapersonal aspect is divided into individual knowledge, attitude, and behavior, the interpersonal aspect is divided into social support that can provide social resources to individuals, and the organization/community factor is divided into various social resources. In the third stage data collection stage, data were collected by writing the main and auxiliary questions based on the categories and codes of the second stage. Step four is the data coding step, where the collected data is read repeatedly, it was found and displayed meaningful statements about health promoting experiences, the indicated passages were grouped according to predetermined categories after individual coding. The last step is the reporting step, and the meaning of each category for health promoting behavior of low-income overweight and obese women was analyzed, reconstructed, and described in text. Afterward, in order to confirm the validity of the essential topic of health promoting behavior, two research participants were presented with the overall described data and went through a participant verification process to check whether it was consistent with their own experiences. In addition, in order to increase the neutrality, which is the credibility of the research, the whole process and analysis results of the research were reviewed by two nursing professors with rich experience in qualitative research, iterative modifications were made to meaningful statements, codes, and categories.

To ensure consistency, two nursing professors with experience in qualitative research were invited to assess the process and structuring of themes and how meanings were derived from the analysis. The researcher made continuous efforts to exclude self-assumptions, preconceived notions, and prejudices in the process of data collection and analysis.

The final category clusters related to the health promoting behaviors of low-income overweight and obese women were classified into “feeling that the body and mind are not healthy” and “difficulty maintaining a healthy lifestyle.”

*Qualitative rigor*: To ensure rigor according to the criteria of Lincoln and Guba [15], for truth value, the researcher presented interview data and analysis results to all 10 participants to obtain agreement on whether the analysis was consistent with the researcher’s understanding. For applicability, the purpose and content of the study, the method of recruiting participants, and the method of data collection and analysis were described in detail.

## Results

### The experience of health promoting behaviors of low-income overweight and obese women

Table 2 shows the results of the content analysis. Six categories were derived, and two category clusters emerged that best describe the experience of health promoting behaviors: despite “feeling that the body and mind are not healthy” they had “difficulty maintaining a healthy lifestyle”. The low-income overweight and obese participants recognized that they had unhealthy health behaviors and that they experienced various limitations in their current health promoting behaviors.

#### 1) Category cluster 1: Feeling that the body and mind are not healthy

The participants in this study were trying to improve their health promoting behaviors gradually every day despite their economic crisis. It is difficult to practice health promoting behavior in a situation that solves various physical, psychological, and social problems. As a result of this study, poor nutrition, lack of physical activity, and high levels of stress were found as intrapersonal level. In addition, poor environment and insufficient use of medical services were explored at the interpersonal level that mutually influences the intrapersonal level. Although participants were classified as low-income and received a certain amount of income through public work and the benefits of governmental support, insufficient money for poor health promoting behavior has always been limited. Thus, economic barriers at the organizational/community level that mutually affect unhealthy were found.

##### Category 1. Intrapersonal level: nutritionally unhealthy, lack of physical activity, and stressful situation

Among participants, the rate of skipping breakfast was high, and many reported not eating well-balanced three meals a day. In particular, vegetables and fruits intake was low, whereas they frequently ate high-carbohydrate diets or fast food. Most of the participants in this study tended to perceive exercise as burdensome because they would have to spend separate time and money on health promoting behaviors, thus they avoided or could not do physical activity. Their main physical activity was walking, which was often during commuting and lunchtime. The participants experienced various stresses such as physical stress-related obesity and psychological stress owing to strained family or relationships. However, coping and management of such stressors were inadequate.

*“Maybe it's because I haven't eaten, so I can't well-balanced meals these days. I don't eat breakfast very well. I eat it simply to get by. I eat one meal a day. I don't eat vegetables and fruits.” (Participant 5; BMI, 24.3 kg/m^2^)*

*"In the evening, though, I eat heartily with delivery food. I like spicy food so much; I eat spicy food and delivery food and eating out is frequent. This is my problem. I usually eat fried chicken and pizza for delivery. I eat a lot of fried chicken. I like to eat late at night.” (Participant 7; BMI, 27.7 kg/m^2^)*

*“I usually go around once during lunchtime. I walk along the riverside every day. I do not do any other physical activities.” (Participant 3; BMI, 30.4 kg/m^2^).*

*“I'm stressed because my husband tells me to lose weight.” “I relieved my stress by eating.” (Participant 8; BMI, 40.6 kg/m^2^)*

*I get stressed at work when people treat me rudely. (Participant 2; BMI, 23.8 kg/m^2^)*

##### Category 2. Interpersonal level: a poor environment and inaccessible medical services

Another factor for feeling that the body and mind are not healthy centered on their environment. Most participants voiced problems with living in a poor environment because there were no parks or sports facilities for physical activity in their neighborhood. For some, their residence was located on a steep hill, which limited their mobility and discouraged health promotion practices. In addition, they experienced difficulties in receiving basic services because they were far from the public health center, which offered primary health care services but was not capable of covering more serious illnesses. Problems with access to the overall medical system were also noted, such as not receiving guidance from existing programs within the healthcare system, e.g., health promoting programs and obesity prevention programs.

*“When I come home from work, I get tired and fall asleep or collapse. The road going up to the house is quite steep, so going up the house is a physical activity. I have never participated in a public health center health program. The health center was too far from my house.” (Participant 6; BMI, 27.4 kg/m^2^)*

*“There are no parks or facilities to exercise near my house.” (Participant 1; BMI, 29.9 kg/m^2^)*

*“I didn't know there was a health promotion program in healthcare center. Where do you get info for that?” (Participant 4; BMI, 35.7 kg/m^2^)*

##### Category 3. Organizational/community level: economic barriers not enough money

Despite some degree of welfare support, most participants were weighed down by the economic burden of being responsible for the family's livelihood. Thus, they had to prioritize surviving over health promoting behaviors. Health promoting behaviors were economic issues “Not having enough money”.

*“I am not financially wealthy. I cannot seem to live well. My family lives on what I earn alone. I have to take care of the children before myself, so I can't even think about physical activity. I thought the kids would die when I fell, so I just did whatever work I could get. It was hard because I had no living money. It's the same even now.” (Participant 3; BMI, 30.4 kg/m^2^)*

#### 2) Category cluster 2: Difficulty maintaining a healthy lifestyle

Participants also shared that it was difficult to practice maintaining health promoting behaviors, and several intrapersonal level, interpersonal level, and organizational/community level were found. Intrapersonal level included an unwillingness or a lack of relevant information, interpersonal level included lack of support system, and organizational/community level included not much time and too much work on seeking and maintaining a healthy lifestyle. Among the factors that made it difficult for participants to maintain health promoting behaviors were a lack of personal will, a lack of information related to health promoting behaviors, a lack of time, and too many overall burdens to earn a living for a family on maintain health promotion behaviors.

##### Category 4. Intrapersonal level: lack of personal will, a lack of information

Participants had poor willpower or lack of willpower to control their weight, resulting in poor physical activity or diet.

However, they desired information on how to incorporate healthy lifestyle patterns more effectively in realistic situations. Although many participants wanted to continue physical activity for weight control and wanted to learn weight control activities other than basic walking, they were frustrated by the lack of concrete and relevant information. Participants also desired information on how to incorporate physical activity more effectively in realistic situations.

*“Last year, I went to the gym for 3 months, and that was the only time I lost weight. The reason I can't keep doing this is that I don't have the will. I have thought that I have to do it if I have to, but it doesn't work well in action. The biggest problem is the lack of willpower.” (Participant 4; BMI, 35.7 kg/m^2^)*

*“I know I need to exercise a lot, but I don't know how to do it. I wish someone could tell me how to exercise.” (Participant 8, BMI; 40.6 kg/m^2^)*

*“I want to exercise. I want to try something other than walking. However, I do not know how to start. I have to work during the day. I don't have accurate medical knowledge, so I want to know about health information.” (Participant 2; BMI, 23.8 kg/m^2^)*

##### Category 5. Interpersonal level: lack of support system

In addition, the participants in this study noted a lack of supportive people that could be inducive for health promotion behaviors. Participants wanted to receive positive feedback from people around them and gain stability from a support system. However, the reality was expressing regret that this was not the case. The absence of a close person to remind them of healthy lifestyle and behaviors discouraged them.

*“There is no such person around. I have to take care of it (e.g. food, clothing and shelter, child support) all by myself.” (Participant 4; BMI, 35.7 kg/m^2^)*

*“It's annoying. Everything is bothering me. If I have someone, I cook, but there's no one by my side. These days, I don't cook, I just scoop it up alone, and it's become chronic. Also, my husband suddenly died, and I had nowhere to rely on, and it was hard. I had a hard time living. It's a little sad that all my family members are separated.” (Participant 5; BMI, 24.3 kg/m^2^)*

##### Category 6. Organizational/community level: not much time and too much work

Health promoting behaviors tended to be viewed as something participants had to make time for in their busy lives. However, they had too much work and thus, no time to spare for maintaining health promotion behaviors.

*“I'm busy taking care of my work. I'm busy with work and housekeeping. I have to set aside time to exercise, but I have so much work to do that it’s hard to find time.” (Participant 3; BMI, 30.4 kg/m^2^)*

*“Life itself is very difficult. My whole family lives on what I earn alone (suddenly crying).” (Participant 3; BMI, 30.4 kg/m^2^)*

## Discussion

This study was conducted to explore and describe how low-income overweight and obese women in Korea experience their health promoting behaviors. The ten participants in this study frequently discussed emotional eating, overeating, and expressed experiences similar to reports in the literature for overweight and obese women of low economic status: nutritional instability [16], high rates of abdominal obesity, and low levels of physical activity [17]. Prior studies have reported that 75.6% of low-income obese women did not engage in aerobic physical activity, 30.4% had depression, and 34.3% said that they perceived stress in daily life [18]. In this study, it was confirmed that the attention of the healthcare providers is necessary to maintain a healthy lifestyle of low-income, overweight and obese women because the participants have an unhealthy lifestyle and experience a lot of stress.

In this regard, developing effective dietary intervention strategies are critical for low-income persons, to overcome patterns of monotonous food consumption and eating too much food with a high glycemic index and lipid content [19]. Time-restricted intake interventions have been shown as a potential strategy for decreasing emotional eating patterns in socially vulnerable low-income obese women in Brazil, which resulted in improvement in waist circumference and body fat [20]. Lifestyle interventions that included nutrition, physical activity, and stress management have also been shown to increase health promoting behaviors among middle-aged low-income Korean women [6,21].

Most of the participants in this study were in a state where they had to take on the role of a householder and make a living, and they experienced a lot of stress in this life. Therefore, spending time and money to exercise regularly or eating healthy food was different from the way of life that had to make a living. The life-course perspective acknowledges that poverty-related socioeconomic problems fuel poor health and, as a result, create cycles that are difficult to break. Nevertheless, the participants wanted changes to maintain a healthy lifestyle. Therefore, it is necessary to enhance health not only personal approaches to nutrition, physical activity, and stress management but also environmental and social approach of low-income obese and overweight women.

The participants in this study mostly acknowledged their unhealthy health promoting behaviors. In this regard, the lack of will, health-related information, and economic power and support system required surrounding help and a support system. They indicated that with such a support system, their health promoting behavior can be improved. Most low-income obese women showed a tendency to do their best in their own environment to engage in health promoting behaviors. Examples noted were attempting to eat a variety of healthy foods, including vegetables, and maintaining physical health through regular walking instead of the gym, which they could not afford. However, it was difficult to maintain these health promoting behaviors by themselves owing to a lack of information. Therefore, it will be necessary to present accurate and detailed healthy lifestyle information, such as explaining the optimum intensity and timing of eating habits and physical activity necessary to maintain health promotion behaviors.

Given that low-income women are affected by the information gap in exploring health-related information, because they often tend to rely on sources that they consider unreliable [22], this study reinforces the need for a support system that can deliver such health-related information in an accurate and accessible way. In addition, as individual, social, and environment-level factors can uniquely influence low-income obese women in setting goals for health promoting behavior, customized strategies for optimal and sustainable change in health promotion behaviors are needed [23]. Low-income overweight and obese women have relatively poor physical and mental health, emphasizing the need to pursue personal, social, and structural strategies to improve their physical and mental health [18]. In the United States, the prevalence of obesity is inversely proportional to the median income of white and black populations, and strategies to increase socioeconomic status have also been influential in reducing obesity [3].

Low-income, overweight and obese women reported that it was difficult to maintain a healthy life due to lack of support from their surroundings in a poor environment where exercise or medical services could not be used properly. In addition, economic barriers and lack of time due to having to work to maintain a livelihood were identified as interpersonal and organizational/community factors that made it difficult to perform health promoting behaviors. The necessity of visiting services to improve the health of these vulnerable people is suggested. Since the visiting service is said to be effective for family and maternal health [24], it will be necessary to develop and apply an effective health management program for individualized health management of the vulnerable. By expanding the scope and scale of the currently implemented government-based visiting service, it is necessary to pay attention to the health management of the vulnerable.

The results of this study can help better inform health care workers who do not recognize various barriers to health promoting behaviors in low-income overweight and obese women [25]. It is worthy to note that 19.4% of low-income overweight and obese women experience unmet medical care and awareness of health conditions of women has been shown to influence unmet health care needs [18].

This study hfocused on low-income overweight and obese women in Korea, a country with national health insurance. Thus transferability to countries that have different health systems is limited. However, findings illustrate specific health promotion issues experienced by low-income overweight and obese women.

In conclusion, the two category clusters of “feeling that the body and mind are not healthy” and “difficulty maintaining a healthy lifestyle” derived from participants suggest that devising and providing a lifestyle intervention that includesdetailed and realistic health-related information is needed for low-income overweight and obese women. Moreover, social support and policies that can alleviate larger socioeconomic barriers to health promotion are needed for these women.

## Figures and Tables

**Table 1.** Demographic characteristics of the participants (N=10)

Participant No.	Age (year)	Body mass index (kg/m^2^)	Monthly family income (Korean won)	Family living together
P1	53	29.9	<1 million	Alone
P2	54	23.8	<1 million	Alone
P3	52	30.4	<1 million	Divorce; 3 children
P4	37	35.7	<1 million	Divorce; 1 child
P5	55	24.3	<1 million	Alone
P6	45	27.4	<1 million	Divorce; 1 child
P7	30	27.7	<1 million	Alone
P8	24	40.6	<1 million	Married; husband, 1 child
P9	23	39.8	<1 million	Alone
P10	53	25.4	<1 million	Married; husband, 3 children

One million Korean won is approximately 900 US dollars.

**Table 2.** Experiences of health promoting behaviors in low-income, overweight and obese women in Korea

Category cluster	Categories
Feeling that the body and mind are not healthy	Intrapersonal level: nutritionally unhealthy, lack of physical activity, and stressful situation
	Interpersonal level: a poor environment and insufficient use of medical services
	Organizational/community level: economic barriers not enough money
Difficulty maintaining a healthy lifestyle	Intrapersonal level: lack of personal will, a lack of information
	Interpersonal level: lack of support system
	Organizational/community level: not much time and too much work
